# Prevalence, demographic factors, co-morbidities, and treatment types associated with acanthosis nigricans in a large cross-section of 900^+^ transmasculine patients: Data from a single academic center 2016–2023

**DOI:** 10.1371/journal.pone.0340907

**Published:** 2026-01-12

**Authors:** Twan Sia, Farah Abou-Taleb, Connie Ma, Shufeng Li, Anne Lynn S. Chang

**Affiliations:** 1 Department of Dermatology, Stanford University School of Medicine, Redwood City, California, United States of America; 2 Department of Urology, Stanford University School of Medicine, Palo Alto, California, United States of America; Kwara State University, NIGERIA

## Abstract

**Background:**

Acanthosis nigricans (AN) is a highly visible cutaneous condition that has been associated with cardiometabolic factors, drugs, or malignancy in various populations. AN can be challenging to treat. Transmasculine patients are a special population, but their AN prevalence, and whether AN associates with demographic factors, co-morbidities and gender affirming care (GAC) treatment types have not been well-studied. Our study explores whether GAC treatment type is associated with AN while controlling for confounders in a large group of transmasculine patients at an academic center.

**Methods:**

Following Institutional Review Board approval, the Stanford Research Repository (01/01/2016–21/09/2023) was searched to identify transmasculine patients for individual chart review. The primary outcome was AN and its association with demographic factors, co-morbidities, and gender-affirming care (GAC) treatment type by multivariate logistic regression (presented as Odds Ratio (OR) and 95% Confidence Interval (CI)).

**Results:**

Out of 945 transmasculine patients, AN prevalence was 4.55%, an elevated rate compared to the overall database prevalence of 0.3% for the same period. Prevalence of AN in transmasculine patients never exposed to GAC was 4.02% (7/174). Median age was 20.1 years (interquartile range (IQR) 17.2–25.4). On multivariate logistic regression, AN was associated with obesity (OR 9.31, 95% CI 4.40–20.33), metabolic syndrome (OR 4.04, 95% CI 1.09–15.23), prediabetes (OR 3.17, 95% CI 1.10–8.54), hypertension (OR 2.74, 95% CI 0.96–7.18), and Hispanic ethnicity (OR 2.46, 95% CI 1.12–5.25). GAC type (including exogenous testosterone usage) was not associated with AN, despite >99% power to detect a 10% difference in AN prevalence.

**Conclusions:**

Because AN is enriched in transmasculine patients, and precedes co-morbidities in a majority of cases, dermatologists and other physicians should consider examination of common areas for AN in in transmasculine patients, as screening and diagnosis of co-morbidities could lead to improved health outcomes.

## Introduction

Acanthosis nigricans (AN) is a highly visible cutaneous condition that can be challenging to treat [1,2]. More importantly, AN has been associated with underlying medical conditions including obesity [3–8], metabolic syndromes [8–10], pre-diabetes [3,11], diabetes mellitus [12–15], hyperlipidemia [4,5], hypertension [6,7,16], malignancy [2], or medication usage in various populations [17,18]. However, the risk of AN and comorbidities in the transmasculine population has not been systematically explored.

AN is associated with elevated androgen levels in specific syndromes. The multisystemic syndrome of hyperandrogenism, insulin resistance, and AN (HAIR-AN) has been described in cis-gendered women [19–21]. Testosterone-induced AN in Klinefelter’s syndrome or adult-onset idiopathic hypogonadotropic hypogonadism patients have been observed [22,23]. In one small study, patients with AN had an increased prevalence of elevated dehydroepiandrosterone sulphate (DHEAS) and serum testosterone [24].

Transgender patients comprise approximately 1% of the United States population, according to the Centers for Disease Control in 2021, with the majority under age 35 years. In a 2016 survey study, a majority of transgender patients noted that a major concern of initiating gender affirming care (GAC) was potential unwanted aesthetic changes [25]. Thus, high quality data on associations with AN, a visible skin change, in transmasculine patients is important for counseling patients [26].

The prevalence, and potential demographic and comorbid associations of AN in transmasculine patients, an under-studied and vulnerable population, have not been well-described. Gender-affirming care (GAC) for transmasculine individuals often involves the use of exogenous testosterone to achieve physiologic male levels of hormones rather than supra-physiologic levels as found in HAIR-AN or Klinefelter’s syndrome. Furthermore, some prior literature has linked exogenous testosterone usage in transmasculine patients with hypertension and hyperlipidemia [27–29], which may associate with AN. However, whether GAC in transmasculine patients associates with AN (while controlling for confounders) is unclear.

Here, we utilize a large cross-sectional database at an academic center to better understand the prevalence, demographic factors, comorbidities, and GAC treatment types associated with AN in transmasculine patients.

## Materials and methods

### Patient cohort

This study, including the waiver of informed patient consent, and the use of the Stanford Research Repository (STARR) was approved by the Stanford Human Subjects Panel (Protocol #70746). Waiver of informed patient consent was approved as “the research involves no more than minimal risk”. This research was completed in accordance with the Declaration of Helsinki as revised in 2013.

The Stanford Research Repository (STARR) is a web-based database which includes electronic health records from Stanford Health Care, Stanford Health Care Tri-Valley, University Healthcare Alliance, and Stanford Medicine Children’s Health (Lucile Packard Children’s Hospital and Packard Children’s Health Alliance). Data from STARR between 01/01/2016 to 21/09/2023 were accessed on 21/09/2023 for research purposes. Data beyond 21/09/2023 were excluded.

The following search terms were applied: “transgender male”, “FTM”, “female-to-male”, “female to male”, “transmale”, “transmasculine”, “trans male”, “trans masculine”, “trans man”, “transman”, “transgender man”, or “transgender male”. Transmasculine patients 8 years or older were included in the study, since data has suggested that >90% of referrals for GAC occur at this age threshold [30]. Individual chart review was performed to confirm that each patient self-identified with a transmasculine gender identity, broadly defined as a people assigned female at birth who are transitioning to an either partially or fully masculine gender identity, regardless of GAC treatment. This methodology was used as terminology has changed over time, including International Classifications of Disease codes [31]. Our study team also acknowledges that some of our search terms are now considered outdated language, however, as they were historically commonly used terms are important to include to capture as many transmasculine patients as possible. Additional search terms applied included the diagnosis of acanthosis nigricans (using International Classification of Diseases, 10^th^ edition (ICD-10) code L83).

### Data extraction

Data from individual chart review was extracted to include demographic information (age at date of data freeze (21/09/2023), race, ethnicity, number of dermatology visits) and comorbidities known to associate with AN in the medical literature (*a priori* to analysis*)*. The comorbidities were defined according to ICD-10 codes in the medical record as assigned by the treating physician, and each chart was individually reviewed to confirm the diagnosis in the text of the physician’s progress notes. ICD-10 codes were the following: obesity (ICD-10 codes E65-E68) [3–8], metabolic syndrome (ICD-10 code E88.81) [8–10], diabetes mellitus (ICD-10 codes E08-E13) [12–15], hyperlipidemia (ICD-10 codes E78.2, E78.4, E78.5) [4,5], pre-diabetes (ICD-10 code R73.03 without E08-E13) [3,11], and hypertension (ICD-10 codes E08-E13) [6,7,16].

GAC exposure types, if any, were recorded and included exogenous testosterone, menstrual suppression (e.g., progestin), pubertal blockers (e.g., gonadotropin-releasing hormone agonists), or oophorectomy. Oophorectomy was included in this study as it could impact sex hormone levels [32]. If testosterone was used, the dosages and durations used were recorded whenever available.

Anatomic location(s) of AN were recorded whenever noted in the medical record.

### Data analysis

Prevalence of AN was calculated by dividing the population at risk for AN into the total number of AN patients for the period detailed above. Descriptive statistics of continuous variables are reported as medians and interquartile ranges, and categorical variables are reported as frequency and percentage. To assess the factors that associate with AN, Mann-Whitney test for continuous variables and Fisher’s exact test for categorical variables were used to compare patients with and without. Odds ratios (ORs) were calculated using the Baptista-Pike method. Multivariate logistic regression was applied to investigate factors significant on univariate analysis. All tests were two-sided. P < 0.05 was considered significant. P-values were corrected (indicated by p_c_) for false discovery due to testing of 10 hypotheses. Analyses were performed using GraphPad Prism 10.4.1 (San Diego, CA) and/or Statistical Analysis System (SAS) Institute Inc., version 9.4, (Cary, NC).

## Results

### Cohort characteristics

Using the aforementioned search terms, 3,488 patients were identified. Individual chart review excluded 2,543 patients as transmasculine search terms appeared in their charts for reasons besides the patient’s gender identity (e.g., family history). Thus, the analysis cohort was 945 patients with confirmed transmasculine gender identity.

**Fig 1:**
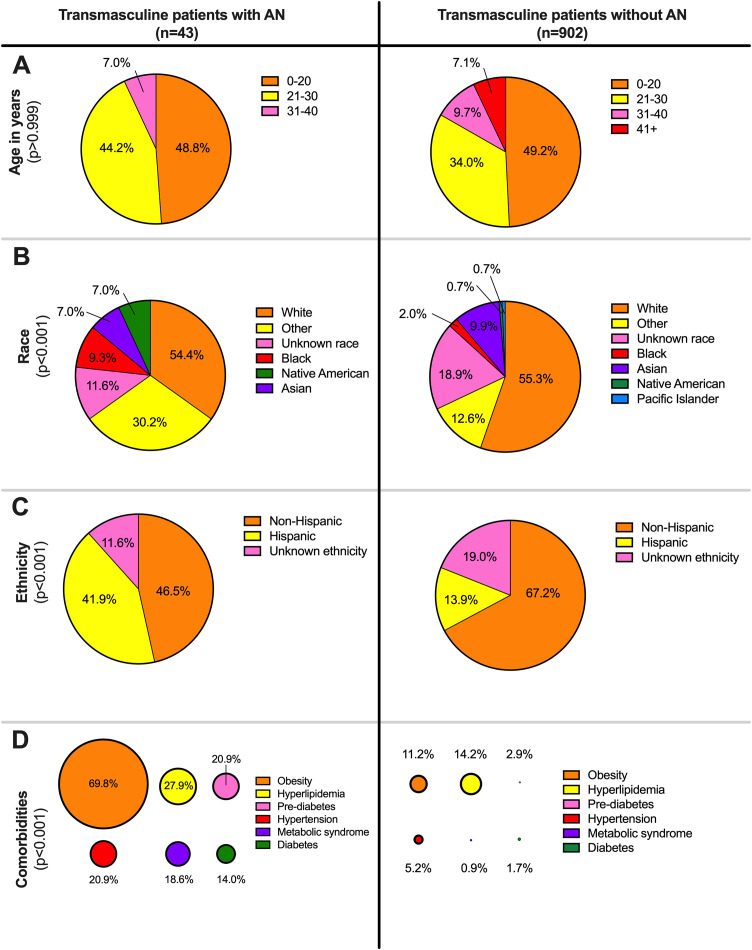
Schematics of demographic and comorbidities in transmasculine patients with and without acanthosis nigricans. (A) Age in years at the date of data freeze (21/09/2023), (B) race, (C), ethnicity, and (D) comorbidities known to associate with AN in literature. P-values were calculated using Mann-Whitney test for age and Fisher’s exact test for race, ethnicity, and comorbidities. P-values were corrected for multiple hypothesis testing (m = 10).

Overall prevalence of AN among transmasculine patients was 4.55% (43/945), an elevated rate compared to the overall database prevalence of 0.3% (8,775/3,555,435) for the same period. Prevalence of AN in transmasculine patients never exposed to GAC was 4.02% (7/174). Of these 945 transmasculine patients, the median age was 20.1 years old (Interquartile Range (IQR) 17.2–25.4) (Table 1) with 33 patients (3.5%) 51 years or older and 151 (16.0%) 15 years old or younger. There were no significant differences in median age between transmasculine patients with and without AN (Fig 1A).

**Table 1:** Demographic and clinical characteristics of all included transmasculine patients in the Stanford Research Repository (STARR) 2016-2023. P-values are corrected (indicated by p_c_) for false discovery rate, between transmasculine patients with and without AN. Significant p values are in bold font (p_c_ < 0.05).

Characteristics	All patients in the database (n = 3,555,435)^1^	All transmasculine patients (n = 945)	Transmasculine patients with AN (n = 43)^3^	Transmasculine patients without AN (n = 902)	p_c_ (between latter two columns)
**Median age, years (IQR)**	5^th^ decade with IQR 3rd-7th decades^2^	20.1 (17.2-25.4)	20.1 (18.1-23.0)	20.1 (17.1-25.5)	>0.999
**Race, n (%)**					**<0.001**
White	1,390,745 (39.1)	514 (54.4)	15 (34.9)	499 (55.3)	0.114
Asian	501,365 (14.1)	92 (9.7)	3 (7.0)	89 (9.9)	>0.999
Black	119,950 (3.4)	22 (2.3)	4 (9.3)	18 (2.0)	0.150
Native American	10,735 (0.3)	9 (1.0)	3 (7.0)	6 (0.7)	0.061
Pacific Islander	NR (<0.1)	6 (0.6)	0 (0)	6 (0.7)	N/C
Other	562,540 (15.8)	127 (13.4)	13 (30.2)	114 (12.6)	**0.043**
Unknown race	942,340 (26.5)	175 (18.5)	5 (11.6)	170 (18.9)	>0.999
**Ethnicity, n (%)**					**<0.001**
Hispanic	473,915 (13.3)	143 (15.1)	18 (41.9)	125 (13.9)	**<0.001**
Non-Hispanic	2,094,705 (58.9)	626 (66.2)	20 (46.5)	606 (67.2)	0.076
Unknown ethnicity	986,820 (27.8)	176 (18.6)	5 (11.6)	171 (19.0)	>0.999
**Dermatology clinic visit (at least one), n (%)**	309,275 (8.7)	162 (17.1)	16 (37.2)	146 (16.2)	**0.013**
**Select comorbidities, n (%)**					**0.002**
Diabetes	176,140 (5.0)	21 (2.2)	6 (14.0)	15 (1.7)	**0.002**
Pre-diabetes without DM	55,125 (1.6)	35 (3.7)	9 (20.9)	26 (2.9)	**<0.001**
Hyperlipidemia	381,300 (10.7)	140 (14.8)	12 (27.9)	128 (14.2)	0.247
Obesity	200,215 (5.6)	131 (13.9)	30 (69.8)	101 (11.2)	**<0.001**
Hypertension	421,380 (11.9)	56 (5.9)	9 (20.9)	47 (5.2)	**0.005**
Metabolic syndrome	8,930 (0.3)	16 (1.7)	8 (18.6)	8 (0.9)	**<0.001**

^1^All numbers in this column rounded to nearest 5 per STARR database. ^2^STARR database provides grouped data only. ^3^Of 43 patients with AN, 20 (46.5%) were diagnosed by endocrinologists, 15 (34.9%) by primary care physicians, 3 (7%) by gastroenterologists, 2 (4.7%) by gynecologists, 1 (2.3%) by a dermatologist, 1 (2.3%) by a cardiologist and 1 (2.3%) by a nephrologist. N/C = not calculated, NR = not reported, AN = acanthosis nigricans, CI = confidence interval, DM = diabetes mellitus IQR = interquartile range, OR=odds ratio.

Most transmasculine patients were White race (n = 514, 54.4%) and non-Hispanic ethnicity (n = 626, 66.2%) (Fig 1B and 1C). Hispanic transmasculine patients comprised 15% (n = 143) of the cohort. The most common comorbidities among all 945 transmasculine patients were hyperlipidemia (n = 140, 14.8%) and obesity (n = 131, 13.9%) (Fig 1D). Of the 945 transmasculine patients, 162 (17.1%) had at least 1 dermatology visit.

Most transmasculine patients pursued at least one type of GAC (n = 771, 81.6%). The two most common GAC types were exogenous testosterone (n = 623, 65.9%) and/or menstrual suppression (n = 370, 39.2%) (Table 2). Median duration of exogenous testosterone usage was 3.98 years (IQR 2.28–6.46, among n = 565 with available data), with most patients achieving a typical maintenance dose of testosterone, as defined in Table 3.

**Table 2:** Usage and types of gender-affirming care (GAC) in all included transmasculine patients (n = 945) in the Stanford Research Repository. None of the gender-affirming treatments when analyzed in aggregate nor individually by treatment type was associated with acanthosis nigricans (AN). Univariate analysis comparing patients with versus without AN was conducted using Fisher’s exact test. All statistic tests are two-tailed. Odds ratios (ORs) were calculated using the Baptista-Pike method. P-values are between transmasculine patients with and without AN.

Treatment^1^	All transmasculine patients (n = 945)	Transmasculine patients with AN (n = 43)	Transmasculine patients without AN (n = 902)	OR (95% CI)	p^2^
Any medical/ surgical gender-affirming care, n (%)	771 (81.6)	36 (83.7)	735 (81.5)	1.17 (0.54-2.76)	0.842
Exogenous testosterone, n (%)	623 (65.9)	26 (60.5)	597 (66.2)	0.78 (0.42-1.45)	0.510
Menstrual suppression, n (%)	370 (39.2)	21 (48.8)	349 (38.7)	1.51 (0.82-2.76)	0.202
Pubertal blockers, n (%)	51 (5.4)	2 (4.7)	49 (5.4)	0.85 (0.20-3.35)	>0.999
Oophorectomy, n (%)	49 (5.2)	2 (4.7)	47 (5.2)	0.89 (0.21-3.51)	>0.999

^1^Gender-affirming care types were not mutually exclusive. ^2^Post-hoc correction for false discovery rate did not impact results. AN = acanthosis nigricans, OR=odds ratio, CI = confidence interval.

**Table 3:** Subset analysis to assess whether testosterone (T) type, dosage, or duration may associate with acanthosis nigricans (AN) in transmasculine patients. There was no significant difference between median years on exogenous T in patients with versus without AN. P-values are for transmasculine patients exposed to T with and without AN.

Characteristics	All transmasculine patients exposed to T (n = 623)	Transmasculine patients exposed to T with AN (n = 26)	Transmasculine patients exposed to T without AN (n = 597)	p-value
Median years on exogenous testosterone (IQR)^1^	4.0 (2.3-6.5)	5.5 (2.3-7.5)	3.9 (2.3-6.4)	0.228
Achieved typical maintenance testosterone dose^2^, n (%)^3^	345/555 (62.2)	15/24 (62.5)	330/531 (62.2)	1
Type of exogenous T				
IM/SQ T cypionate	464 (74.5)	19 (73.1)	445 (74.5)	0.821
IM/SQ T enanthate	16 (2.6)	0 (0)	16 (2.7)	1
IM/SQ multiple formulations	4 (0.6)	0 (0)	4 (0.7)	1
Transdermal T gel	63 (10.1)	2 (7.7)	61 (10.2)	1
Transdermal T patch	6 (1.0)	0 (0)	6 (1.0)	1
Oral T	1 (0.2)	0 (0)	1 (0.2)	1
Implant T long-acting pellet	1 (0.2)	0 (0)	1 (0.2)	1
Switched/multiple routes of administration	50 (8.0)	5 (19.2)	45 (7.5)	**0.049**
Unknown	18 (2.9)	0 (0)	18 (3.0)	1

^1^Time on exogenous testosterone (T) is unavailable for 58 patients (1 with AN and 57 without), thus were excluding from descriptive statistics. Of 25 patients on T with AN where data were available, 12 were diagnosed with AN before T and 13 were diagnosed after (6 were diagnosed within 1 year of T, 3 between 1–3 years on T, 2 between 3–5 years on T, and 2 after >5 years on T). ^2^Typical T maintenance dose was considered 50 mg weekly for subcutaneous injection, 50 mg daily for topical gel, and 4 mg daily for transdermal patch. ^3^Dosing schedule was unavailable for 68 patients (2 with AN and 66 without), thus were excluded in comparison. Though there were significantly greater patients who switched routes of T administration among patients with AN compared to without, no patients changed T administration due to AN. Significant p values are in bold font (p < 0.05). IM = intramuscular. SQ = subcutaneous.

Diagnosis of AN was first made by an endocrinologist in 46% (n = 20) of cases, followed by the primary care provider (including pediatrician) in 34.9% (n = 15) of cases. Only 1 patient (2.3% of AN cases) was first diagnosed with AN by a dermatologist (Table 1). Among the 42 patients with AN who were not first diagnosed by a dermatologist, 15 (35.7%) had seen a dermatologist, though it is unclear if AN was present at the time of the dermatology visit due to lack of specific documentation of AN in the progress note.

Anatomic location of AN was documented in 32 of 43 transmasculine patient charts. AN was noted on only 1 anatomic site on physical examination in 68.8% (22/32) of patients with AN. The most common anatomic sites for AN (in descending order) were neck (28/32, 87.5%), axilla (8/32, 25%), inguinal (3/32, 9.4%), upper extremities (3/32, 9.4%), and lower extremities (2/32, 6.3%). Of all transmasculine patients with AN, anatomic distribution of AN was not different based on testosterone exposure (proportion exposed to testosterone: neck 16/28, 57.1%; axilla 4/8, 50%; inguinal 2/3, 66.6%; upper extremities 3/3, 100%; and lower extremities 1/2, 50; Fisher’s exact test p-p = 1). None of the patients received treatment for AN as indicated by the progress notes.

### Univariate analyses of demographic factors and comorbidities for association with AN

Univariate analysis between transmasculine patients with and without AN were not significantly different with respect to median age (Table 1). However, transmasculine patients with and without AN demonstrated significant differences in race (p_c_ < 0.001) and ethnicity (p_c_ < 0.001), with Hispanic ethnicity demonstrating increased rate of AN.

Of the co-morbidities, obesity, metabolic syndrome, diabetes, pre-diabetes and hypertension were associated in univariate analysis with AN in transmasculine patients. Hyperlipidemia was not associated with AN in transmasculine patients (Table 1).

Transmasculine patients who had been seen by a dermatologist at least once were more likely to associate with an AN diagnosis (OR 3.07, 95% CI 1.58–5.69; p_c_ = 0.013) (Table 1).

Among transmasculine patients, AN was not associated with any GAC or GAC type (e.g., any medical/surgical GAC, exogenous testosterone, menstrual suppression, puberty blockers, or oophorectomy; all with p > 0.05) (Table 2), despite >99% power to detect a 10% difference in AN between transmasculine patients on versus not on testosterone. Our power to detect an association was calculated using a chi-squared test for two independent sample proportions. The true rate difference of 10% was selected *a priori* as a “clinically meaningful” difference based on previous studies in skin diseases (as there is no clear defined clinically meaningful difference in AN with GAC usage currently in the literature) [33]. AN was also not associated with duration of exogenous testosterone usage (median testosterone usage 5.5 years, IQR 2.3–7.5 in those with AN; median 3.9, IQR 2.3–6.4 in those without AN; p = 0.228) (Table 3). There was no significant difference in the proportion of transmasculine patients who had achieved typical maintenance dose of testosterone between those with and without AN (Table 3). Exogenous testosterone type was also examined, with intramuscular/subcutaneous testosterone cypionate and transdermal testosterone gel, not significantly associated with AN, and all other exogenous testosterone types not associated with any patients with AN (Table 3). Though switching testosterone type/administration route was significantly associated with AN (OR 2.92, 95% CI 1.15–7.82), none were due to development of AN or related comorbidities on individual chart review (Table 3). We hypothesize that the lack of AN may be that the exogenous testosterone doses used in GAC achieves physiologic testosterone levels of males rather than supra-physiologic levels that are found in some syndromes. Likely there are more factors at play that are still unclear.

### Univariate analysis of co-morbidities associated with exogenous testosterone usage unrelated to AN

Since the most common GAC type was testosterone in transmasculine patients (n = 623; 65.9% of patients on GAC), we examined co-morbidities associated with testosterone exposure by univariate analysis and found that hypertension (OR 2.20, 95% CI 1.15–4.25, p = 0.020) and hyperlipidemia (OR 2.19, 95% CI 1.44–3.40, p < 0.001. Both comorbidities have been identified as associated with testosterone usage in prior literature in both transgender [27–29] and cisgender patients [34].

### Multivariate analysis of demographic factors and co-morbidities for association with AN

Multivariate logistic regression of demographic factors and co-morbidities that were significantly associated with AN (regardless of any gender affirming care) in univariate analysis. Hispanic ethnicity was associated with AN (OR 2.46, 95% CI 1.12–5.25; p = 0.022). The following co-morbidities were associated with AN in transmasculine patients (in descending order of OR): obesity (OR 9.31, 95% CI 4.40–20.33; p < 0.001), metabolic syndrome (OR 4.04, 95% CI 1.09–15.23; p = 0.036), pre-diabetes (OR 3.17, 95% CI 1.10–8.54, p = 0.026), and hypertension (OR 2.74, 95% CI 0.96–7.18; p = 0.048) (Table 4).

**Table 4:** Multivariate logistic regression analysis of clinically relevant factors associated with acanthosis nigricans (AN) (N = 945) in transmasculine patients regardless of gender affirming care. Gender-affirming care was not a variable in the model as it was not correlated with AN in univariate analysis (Table 2) or subset analysis (Table 3). Significant p values are in bold font.

Characteristics	OR (95% CI)	p-value
Race		
White	1 (Reference)	
Non-White	1.68 (0.82-3.54)	0.162
Ethnicity		
Hispanic	2.46 (1.12-5.25)	**0.022**
Non-Hispanic	1 (Reference)	
Diabetes status		
No diabetes	1 (Reference)	
Pre-diabetes	3.17 (1.10-8.54)	**0.026**
Diabetes mellitus	1.67 (0.40-6.23)	0.456
Obesity	9.31 (4.40-20.33)	**<0.001**
No obesity	1 (Reference)	
Hypertension	2.74 (0.96-7.18)	**0.048**
No hypertension	1 (Reference)	
Metabolic syndrome	4.04 (1.09-15.23)	**0.036**
No metabolic syndrome	1 (Reference)	

CI=Confidence interval. OR=odds ratio.

### Timing of AN compared to comorbidities’ diagnosis among transmasculine patients

Of 43 transmasculine patients with AN, 34 (79.1%) had at least 1 comorbidity. AN diagnosis preceded all cases of metabolic syndrome in our transmasculine cohort (8/8, 100%). AN was identified prior to diagnosis of comorbidity in a majority of transmasculine patients for hypertension (5/9, 55.6%); diabetes (4/6, 66.7%), prediabetes (6/9, 66.7%). AN diagnosis was approximately equally distributed between pre-and post-diagnosis of comorbidities of obesity and hyperlipidemia (Table 5).

**Table 5:** Among transmasculine patients with acanthosis nigricans (n = 43), AN appeared before, concurrently, or after diagnosis of co-morbidities. Some patients had multiple of these comorbidities and thus are represented in multiple columns.

Timing of AN finding, n (%)	Patients with AN and HTN (n = 9)	Patients with AN and DM (n = 6)	Patients with AN and pre-diabetes without DM (n = 9)	Patients with AN and HLD (n = 12)	Patients with AN and obesity (n = 30)	Patients with AN and MetS (n = 8)
AN identified prior to comorbidity diagnosis	5 (55.6)	4 (66.7)	6 (66.7)	5 (41.7)	12 (40)	8 (100)
AN identified at the same time of comorbidity diagnosis	1 (11.1)	1 (16.7)	1 (11.1)	2 (16.7)	4 (13.3)	0 (0)
AN identified after comorbidity diagnosis	3 (33.3)	1 (16.7)	2 (22.2)	5 (41.7)	14 (46.7)	0 (0)

AN = acanthosis nigricans, HTN = hypertension, DM = diabetes mellitus, HLD = hyperlipidemia, MetS = metabolic syndrome.

## Discussion

Our study presents clinically useful data from a large database with long follow-up times to better care for transmasculine patients. Literature search on PubMed and Embase (accessed October 21, 2025) indicates AN and association with co-morbidities specifically in the transmasculine population has not been previously elucidated. Based on our data, screening for AN may facilitate primary prevention or early diagnosis and management of these diseases to avoid adverse long-term health outcomes from late diagnosis.

Since we found Hispanic transmasculine patients have increased risk of AN, a lower threshold for physical examination in areas commonly affected by AN should be considered for this group. The relationship between AN and Hispanic ethnicity in our study is likely multifactorial, based on previous studies, though these were not specifically in transgender patients. Family studies in Hispanic families identified that AN is highly heritable, suggesting that genetics play a role [35]. AN is also significantly associated with poor eating behaviors in Hispanic children, suggesting that there may also be a social or behavioral basis [35]. Further research in transmasculine patients of Hispanic ethnicity may shed light on these factors.

The percentage of Black transmasculine patients with AN is surprisingly higher than those without (9.3 versus 2.0%), but this was not statistically significant, possibly due to small sample size (n = 22). Future studies with larger numbers of Black transmasculine patients may lend insight into our finding.

We did not find any GAC type associated with AN. However, the most common GAC type in our transmasculine cohort was exogenous testosterone, and its usage was associated with hypertension (OR 2.20, 95% CI 1.15–4.25) and hyperlipidemia (OR 2.19, 95% CI 1.44–3.40), *unrelated to AN*. Thus, despite the finding that testosterone exposure alone was not associated with AN, screening for hypertension and hyperlipidemia is reasonable in transmasculine patients using exogenous testosterone.

Surprisingly, we found the prevalence of AN in transmasculine patients was elevated at 4.55%, compared to the overall database prevalence of 0.3% (8,775/3,555,435) for the same period. In addition, the prevalence of AN in transmasculine patients who had never been exposed to GAC (4.02%) suggested that AN was not drug-induced in our study population. Individual chart review of patients with AN exposed to exogenous testosterone confirmed that AN was not suspected to be drug-induced by the treating physician, as has been reported in cases of exogenous testosterone used for adult-onset idiopathic hypogonadotropic hypogonadism and Klinefelter syndrome [22,23].

Further investigation is needed to understand *why* the AN prevalence in the transmasculine population is elevated (including those without GAC exposure) compared to the AN prevalence in the overall database population. Since the prevalence of AN in transmasculine patients who have never used GAC is elevated, one hypothesis could be genetic factors. For instance, AN is linked to mutations in different genes such as fibroblast growth factor receptor (FGFR) 3 [36,37], peroxisome proliferator activated receptor gamma (PPARγ) [38], or insulin receptor (INSR) [39]. The latter two genes associate with obesity, diabetes and/or endocrine changes, which were clinical associations found in our transmasculine population. Currently, we do not have data to support a connection between genetics and AN in our transmasculine population, but this could be a direction of future research [40,41]. Social and behavioral factors may also be implicated in the high prevalence of AN in transmasculine patients. Physical activity in transgender patients is influenced by gender congruence, minority stress, and discrimination [42,43]. Furthermore, transgender patients have high levels of food insecurity and poor diet, which can also contribute to the development of AN and its metabolic comorbidities [44].

A previous single-center study noted that AN was among the three most common chief complaints for transgender patients in a Hispanic patient population [45]. However, the authors noted that transmasculine patients with AN did not have obesity or prediabetes, implying heterogeneity in the causes of AN [45]. Indeed, this is consistent with the observation that AN can have multiple etiologies besides metabolic cause, as alluded to above, such as syndromic, drug-induced or malignant AN. In our White-majority transmasculine study population, AN was associated with obesity and diabetes, a finding that is consistent with prior studies that examined white populations separately from other races [46].

Our patient cohort is relatively young (median 20.1 years of age), in-line with previous data that GAC is typically initiated in early adulthood [47–49]. Notably, our cohort did not have any transmasculine patients with AN over 40 years old (possibly due to a low number of transmasculine patients over 40 years old [n = 64] and low AN prevalence of 4.55% in our cohort), limiting generalizability to older transmasculine patients. The causes for this low median age are unclear, since obesity and insulin resistance increase with age in the general population. One possibility is that the hormone treatment protocols at our academic center could impact our findings. Further investigation into transmasculine cohorts with older transmasculine patients at other centers are needed.

By far, most common anatomic site of AN in our transmasculine population was the neck, followed by the axillae. In prospective studies, AN has been reported as more common in axillary folds than the neck [4], and this difference could be due to more comprehensive skin examinations than in our retrospective study which relied on standard-of-care examinations in the medical record [11,50].

**Fig 2:**
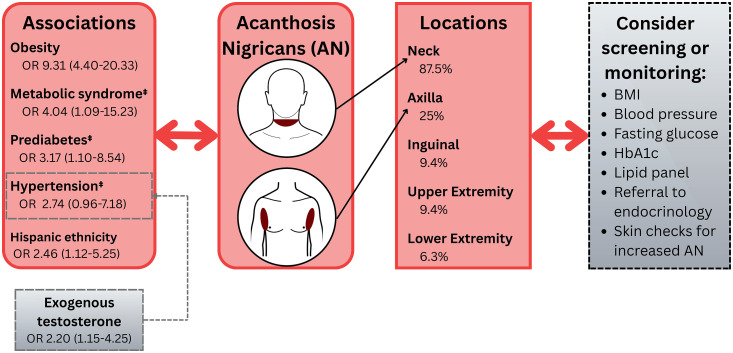
Graphical abstract of study findings and suggestions for screening and monitoring. Odds ratio (OR) and 95% confidence intervals (as displayed in parentheses) were determined by multivariate logistic regression for Hispanic ethnicity, obesity, metabolic syndrome, prediabetes, and hypertension. Univariate analysis was conducted for association between exogenous testosterone and hypertension. Double daggers indicates that in majority of cases, acanthosis nigricans was diagnosed before comorbidities. BMI = Body Mass Index. HbA1c=Hemoglobin A1C. OR=Odds Ratio.

Notably, only 1 patient (2.3%) was diagnosed with AN by a dermatologist, compared to 20 patients (46.5%) by endocrinologists and 15 patients (34.9%) by primary care physicians. Reasons for this are unclear, but could be due to reduced access to dermatologists compared to other physician types, and/or gaps in awareness to check for AN by dermatologists caring for transmasculine patients. To address this potential gap, we have highlighted important considerations for screening transmasculine patients and interdisciplinary care referrals in Fig 2. Educational efforts to dermatologists on increased risks for co-morbidities could also be considered. On individual chart review, none of the 43 transmasculine patients with AN had documented lifestyle, topical, or systemic treatments specifically for their AN. Whether transmasculine patients are less likely to be offered or adopt management options compared to non-transmasculine patients requires further investigation.

Limitations of our work include underestimation of the transmasculine population if this gender identity was not disclosed in the medical record, underestimation of AN prevalence as full body skin examinations were not performed in all included patients, and the lack of specificity in racial subtypes (such as Asians, which pools East, South and Southeast Asians) with their differing skin types and genetics. This study was conducted at a single academic center; therefore, there is the potential for selection bias for our cohort (e.g., socioeconomic factors, access to healthcare, geographical location). Our academic center may also utilize different practices in timing of GAC initiation and/or forms of care compared to other institutions.

In conclusion, our data demonstrate greater prevalence of AN in transmasculine patients regardless of GAC or GAC types in a predominantly white population. In transmasculine patients, AN was associated with Hispanic ethnicity, obesity, metabolic syndrome, prediabetes, and hypertension. We recommend routinely examining the skin (particularly the neck) for AN in transmasculine patients particularly in patients with these risk factors. In addition, calculation of body mass index and monitoring of laboratory values (e.g., fasting glucose, hemoglobin A1C and fasting lipids) can be considered. While hypertension was associated with AN in multivariate analysis in our transmasculine population, GAC and GAC type were not associated with AN. Hence, screening for hypertension with blood pressure checks in transmasculine patients with AN and/or exogenous testosterone usage could be considered. Referrals to endocrinologists and skin checks to monitor for AN exacerbation may also be appropriate (Fig 2). Further research into environmental and lifestyle factors is needed to develop ideal screening guidelines for transmasculine patients with AN.
